# Transdermal delivery of micronutrients through fortified body oil and cosmetics: a potential roadmap for future scale up

**DOI:** 10.3389/fpubh.2023.1136912

**Published:** 2023-06-20

**Authors:** Aditi Apte, Jayeeta Pahan

**Affiliations:** ^1^Vadu Rural Health Program, King Edward Memorial (KEM) Hospital Research Centre, Pune, India; ^2^Murli Krishna Pharma Pvt Ltd, Pune, India

**Keywords:** measurement for change, nutrition, infants, public health, anemia

## 1. Introduction

The initial challenge was to address micronutrient deficiency in early childhood, an important determinant of poor neurocognitive development (1, 2). The innovation selected for transdermal delivery of micronutrients has been scaled up from the laboratory level into clinical trials. It has now received a buy-in from the industry as well as from public health programs and the next challenge is to ensure a continuous and sustainable scaling pathway.

Although iron and folate oral supplementation in under-five children is the strategy adopted by public health programs in India, poor absorption of iron and gastrointestinal adverse effects associated with oral iron are drawbacks associated with the oral supplements leading to suboptimal adherence to the supplements (3, 4). Transdermal drug delivery is associated with peculiar advantages as non-invasive nature of delivery, improved compliance and avoidance of first pass metabolism as well as gastrointestinal irritation (5). We developed an innovative intervention in the form of a liposomal encapsulated micronutrient fortified (LMF) oil for supplementation of iron, vitamin B_12_, folate, and vitamin D which can deliver micronutrients transdermally. The journey of the development of the intervention has been described earlier using a theory of change approach which reflects how the intervention of LMF oil was modified based on the learnings at each step (6). The intervention was evaluated in a community-based randomized controlled study which showed benefit in improving vitamin D levels and partial efficacy in improving anemia (7).

Despite the mixed results of the community-based research study, the intervention received good acceptability in the community. Importantly, there is keen interest and buy-in from the industry partner involved in the clinical trial scale-up. We describe here a potential road map for evaluating this innovation further and scaling it up in the public health program using a theory of change approach.

## 2. Challenges faced so far and lessons learnt

The earlier work focused on translating a laboratory-level innovation in clinical trials through interinstitutional academic collaboration between KEM Hospital Research Centre, Pune, India, and Indian Institute of Technology Bombay, Mumbai, India. Currently, the technology is out-licensed to an industry partner, Murli Krishna Pharma Pvt Ltd, Ranjangaon, Pune. While moving from academic research settings to more commercial and program environments, we need to understand the role of additional stakeholders in the entire process—industry, regulator, and public health bodies. Considering the existing Indian regulations (8) for new drug development, there is a need for comprehensive scientific evidence on this innovation through translational studies. The transdermal route of intervention is an innovative route of supplementation. The extent of transdermal absorption depends upon several factors such as molecular size, lipo or hydrophilicity of the molecule, type of encapsulation, thickness of the skin and use of any penetration enhancers (5). There is limited information available about the exact mechanism of absorption of the ingredients from the skin (5). The extent of absorption in children cannot be completely predicted from animal studies as animal skin is different from human skin. Additionally, the formulation of the LMF oil may need changes to improve the shelf stability and ability to incorporate higher doses of micronutrients, especially iron. Due to these factors, more rigorous standardization and clinical evaluation of the intervention are needed before scaling it up.

## 3. Translation of the innovation—Theory of change

**Figure 1:**
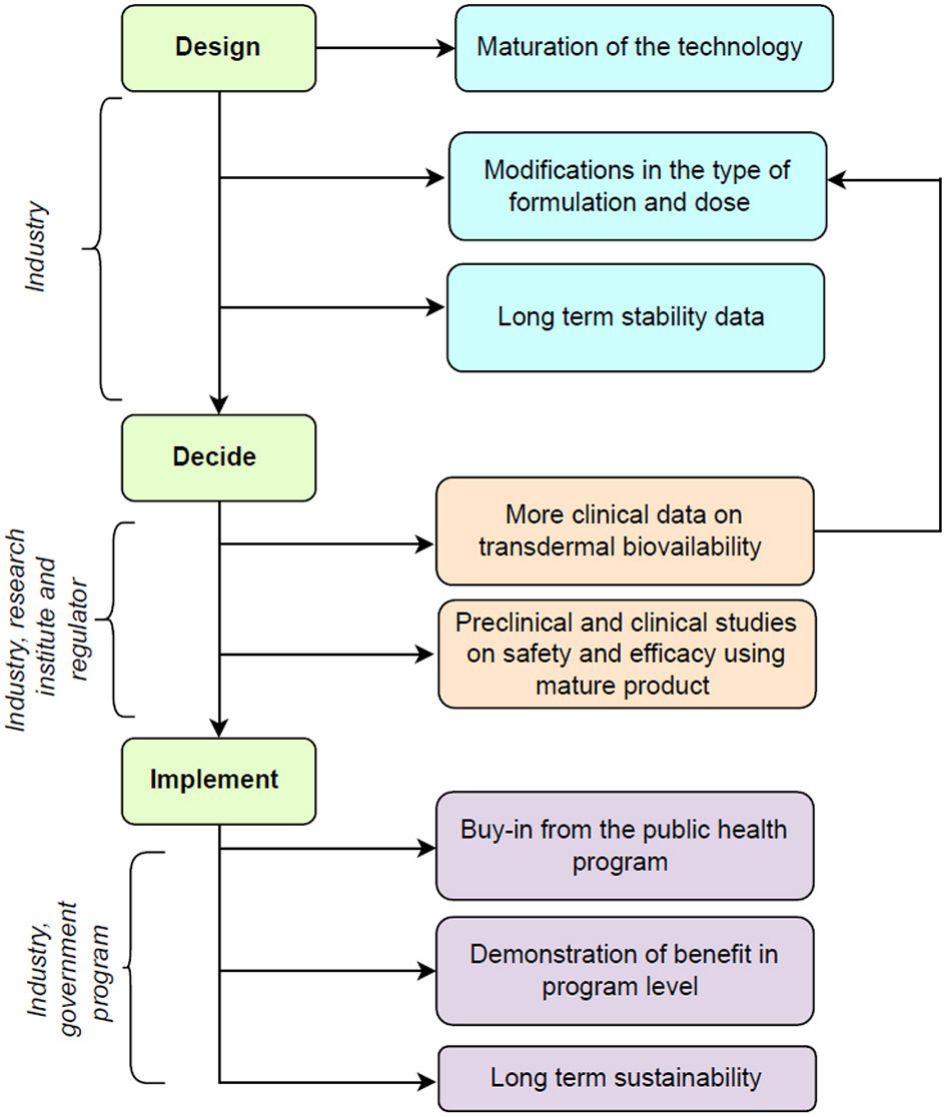
Potential road map for future translation of technology for transdermal micronutrient delivery.

A theory of change framework can help to integrate the monitoring, evaluation, and learning into the decision-making process while transitioning from clinical research to program implementation of this intervention (9). Figure 1 shows the potential roadmap for this innovation and includes the following phases.

### 3.1. Design—Innovative application of previous knowledge

Lipid nanostructure can be a useful platform for the transdermal delivery of micronutrients as they can reversibly fluidize the stratum corneum to deliver the entrapped substances into the deeper dermis and can be synthesized using food-grade materials that are safe for human use (10). The previous study pilot-translated the technology for encapsulation of iron, folic acid, vitamin B_12_, and vitamin D and its incorporation in body oil from a bench-level technology to clinical trial settings using methods that did not involve any organic solvent (5). The technology used was frugal and thus can be affordable for larger public use. The future work will involve scaling it up by the industry partner further for large-scale production. This will involve the use of lyophilization methods for long-term preservation and better stability of the liposomes and refining of the manufacturing processes to deliver purely encapsulated micronutrients in liposomes, to avoid any residual free actives on the skin. Also, to incorporate higher doses of micronutrients, the exploration of alternate formulations in the form of lotions and creams is planned.

### 3.2. Decide—Answers and more questions

The technology has demonstrated safety and efficacy in *in vitro* and *in vivo* animal models for transdermal delivery of nutrients at supplemental doses (Supplementary Table 1). The technology has also demonstrated no irritation potential during irritation patch testing in healthy human volunteers and showed good acceptability in terms of texture, odor, color, consistency and ease of use (6). In future, industry-academic collaboration is required to generate more evidence on the transdermal penetration of micronutrients, i.e., iron, vitamin B_12_, folate, and vitamin D. Evidence from the completed study shows there is scope for increasing the amounts of ferrous bisglycinate and vitamin D as the presently included doses resulted in partial efficacy, however, were safe to administer especially in young infants (7). Modification of the dose will require clear evidence of efficacy and safety from pharmacokinetic studies in animals and humans. Additionally, we plan to utilize the bio-samples of the earlier study to evaluate the changes in the vitamin B_12_ and folate levels. It is known that iron uptake may be better in children with iron deficiency as compared to those without (11). Estimation of the bio-samples for specific biomarkers for iron deficiency i.e., ferritin is the next step to understand whether the intervention works betters in iron-deficient children than non-iron-deficient children. In addition, there is scope for conducting transdermal bioavailability studies on each of the micronutrients using stable isotope techniques in adults and children that allows for accurate measurement of the bioavailability of micronutrients (12). These studies will help in understanding the dose-response relations and can help to finalize the dose to be incorporated into the unit volume of the formulation. The previous study focused on the prevention of micronutrient deficiencies and used doses less than recommended dietary intakes for the given micronutrients. The intervention needs to be evaluated using higher doses and in therapeutic studies for the treatment of anemia and micronutrient deficiencies where pivotal studies may need regulatory approvals.

### 3.3. Implement—Partnerships and networks

The intervention has received a buy-in from the Anemia Mukt Bharat (AMB) which is a public health program by the Government of India for the prevention of anemia in children and pregnant women (3). Government of India's ease of doing business (EODB) initiative to transform healthcare in India through technology and the latest innovations in the field of healthcare (13). The purpose is to envision and develop sustainable models in the health tech sector to significantly improve access, quality of care, and efficiency. The industry partner will be participating in the EODB initiative to scale up this innovation to the AMB program beneficiaries in a stepwise manner. This will involve a demonstration of program implementation at the district/state level followed by implementation over several states. The data gathered from the clinical efficacy studies will help in refining the intervention further before large-scale implementation.

## 4. Summary

The innovation for transdermal delivery of micronutrients has successfully transitioned from an idea into clinical trials, receiving support from industry and public health systems. Further development and translation of this innovation will depend upon continued buy-in from multiple partners, which in turn will depend upon generation of more scientific evidence on efficacy and safety. In structuring a process of continuous learning, we consider the need for monitoring and evaluation to be dynamic and inclusive to generate knowledge about the effectiveness of the intervention followed by effective delivery and scale-up.

## Author contributions

AA conceptualized the idea, drafted the framework for M4C, and finalized the manuscript after review from the M4C reviewers. AA and JP drafted the manuscript. Both authors contributed to the article and approved the submitted version.
