# Supplementary material for: Transdermal delivery of micronutrients through fortified body oil and cosmetics: a potential roadmap for future scale up
Source: Front Public Health. 2023 Jun 20;11:1136912. doi: 10.3389/fpubh.2023.1136912 (PMC10318147; doi:10.3389/fpubh.2023.1136912)
Supplement: Supplementary file 1 [file Table_1.DOCX]

**Supplemementary table 1 - Summary of in vitro and in vivo studies conducted on LMF oil**

| **1. Characterization of micronutrient liposomes (average values)** | | **2. Composition of LMF oil (per 2.5 ml)** | |
| --- | --- | --- | --- |
| Hydrodynamic size (nm) | 161.25 | Ferrous bisglycinate equivalent to elemental iron | 1-1.5 mg |
| Polydispersity index | 0.117 | Folic acid | 40 mcg |
| Zeta potential (mV) | -29.8 | Cyanocobalamin | 0.3 mcg |
| ***Encapsulation efficiency (%)*** | | Cholecalciferol | 400 IU |
| Iron | 55.39 + 2.48 | **3. Preclinical toxicity studies** | **Doses used** |
| Vitamin D | 97 + 1.5 | Nontoxic in subacute dermal toxicity study in Wistar rats and New Zealand rabbits | 1x, 3x and 5x doses of LMF oil applied once daily for 28 days |
| Folic acid | 42.16 + 2.35 | **4. Human tolerability studies** | **Dose used** |
| Vitamin B_12_ | 26.2 + 1.86 | Non-irritant in 26 adult healthy volunteers as compared to positive control | 1x single dose applied over 24 hrs |
| **5. Acceptability of LMF body oil in children** | | Nonirritant in 15 adult healthy volunteers | 1x multiple doses over 15 days |
| Acceptability score 28 out of 30 in terms of appearance, texture, fragrance, ease of use, irritation, and overall evaluation | | Non-irritant in 15 healthy children under two years of age | 1x multiple doses over 15 days |
| **6. Safety and efficacy in infants in a randomized placebo controlled study** | | | |
| - Total 444 children randomized to receive LMF oil or placebo from 4-6 weeks to 12 months of age - Significant improvement in 25-OH-D at 12 months in the LMF oil group [+1.46vs.-0.18ng/ml,p=0.049] - In the subgroup of infants with moderate anemia, the intervention prevented decline in hemoglobin at 12 months of age [adjusted mean change +0.11vs.-0.51gm%,p=0.043]. - Marginal improvement in vitamin B_12_ in a subsample - No overall improvement in hemoglobin levels or developmental scores - No significant increase in systemic or local adverse events | | | |
